# Missing Medical Data in Neurological Emergency Care Compromise Patient Safety and Healthcare Resources

**DOI:** 10.3390/jcm13216344

**Published:** 2024-10-23

**Authors:** Lea Krey, Ziad Rabea, Olaf Krause, Stephan Greten, Johannes Heck, Anna-Lena Boeck, Susanne Petri, Florian Wegner, Martin Klietz

**Affiliations:** 1 Department of Neurology, Hannover Medical School, Carl-Neuberg-Straße 1, 30625 Hannover, Germany; ziad.rabea@stud.mh-hannover.de (Z.R.); klietz.martin@mh-hannover.de (M.K.); 2 Department of General Medicine and Palliative Care, Center for Medicine of the Elderly, Hannover Medical School, Carl-Neuberg-Straße 1, 30625 Hannover, Germany; 3 Center for Geriatric Medicine, Hospital DIAKOVERE Henriettenstift, Schwemannstrasse 19, 30559 Hannover, Germany; 4 Institute for Clinical Pharmacology, Hannover Medical School, Carl-Neuberg-Straße 1, 30625 Hannover, Germany

**Keywords:** patient safety, medical data, emergency care, polypharmacy, drug safety

## Abstract

**Background:** Acute care of patients in the emergency department (ED) can be very challenging when patients attend EDs without their important medical information. This is especially problematic for multimorbid patients under polypharmacy. The aim of this study was to assess systematically the frequency and clinical relevance of incomplete medical data upon ED admission. **Methods:** The study was conducted in the neurological ED of a German tertiary hospital. The availability and accuracy of medical data of all neurological patients in the ED were assessed upon arrival. Treating ED physicians were asked about the acute care of the patients to clarify whether missing data resulted in delays or complications in the emergency treatment. Additionally, doctors responsible for the inpatient care of patients who were admitted to a ward via the ED were questioned about the course of the inpatient stay to monitor how initially missing data might have influenced the hospital stay. **Results:** Medical data of 27% of the 272 included patients were missing or incomplete upon admission in the ED. The ED physicians had to make additional phone calls to gather information in 57% of these cases (vs. 22% in patients with complete data, *p* < 0.0001). Delays between 5 and 240 min were documented due to initially missing data. Unnecessary diagnostic procedures (e.g., lumbar puncture) were performed in 5% of these patients, thus compromising patient safety. Even the inpatient stay was complicated by initially missing data, as doctors still had to spend time (between 10 and 180 min) to gain relevant information. Retrospectively, 5% of hospitalizations could have been avoided if all medical information had been available upon ED admission. **Conclusions:** Missing medical data caused complications and delays in acute as well as inpatient care of patients admitted to the neurological ED. This compromised patient safety and led to a waste of medical resources and valuable time of the responsible medical team. Therefore, a comprehensive, digital data management system is urgently needed to improve patient safety and facilitate efficient patient care in the ED and beyond.

## 1. Introduction

During the assessment of patients in the emergency department (ED), time is a critical resource and the medical history of patients can be crucial in deciding about potential diagnostic procedures or treatment options in an individual case. Having complete medical data upon ED admission is important to achieve quick and appropriate decisions in acute care. This is especially relevant for multimorbid patients with a complex medical history that has to be considered when making these decisions [1]. Multimorbidity has a very high prevalence of 39.2% in the overall population in Europe [2]. There will be even more multimorbid patients receiving polypharmacy in the future due to demographic developments [2,3,4]. These patients are expected to be especially vulnerable to treatment-associated complications [3,5].

In a previous pilot study, we found that medical data were missing for 27.4% of geriatric patients upon admission to the neurological ED [6]. We observed that this resulted in potentially dangerous scenarios for the patients, like delays in treatment, or led to treatments despite relevant contraindications [6].

Based on these data, we hypothesized that incomplete medical data complicate the acute care of patients in the neurological ED. This can hypothetically have an impact on different patient-associated factors. Most importantly, patient safety can be compromised if misjudgments occur and inadequate diagnostic procedures or therapeutic interventions are applied. Apart from that, patients’ wishes can be involuntarily disrespected and patient autonomy can be reduced if data on medical history and patients’ wishes are missing.

Furthermore, doctors may need to spend significant time gathering important information, causing delays in acute and inpatient care. If doctors need to spend valuable time acquiring information about the patient, less time is available for other interactions (e.g., a detailed dialogue with the patient).

Another dimension of this topic is the economic impact on the healthcare system. Unnecessary diagnostic procedures, treatments or hospital admissions that could occur due to missing information result in a potentially avoidable financial burden [7,8,9]. A digital data management system for medical information could be a solution to the suspected problems.

The aim of this study was to take a closer look at the availability of medical data of all neurological patients upon their admission to the ED of a German university clinic and to investigate the consequences of missing data for acute treatment as well as the following inpatient care. We highlight patient safety issues, the aspect of patient autonomy regarding decisions in acute and inpatient care, and investigate how missing data might lead to higher healthcare costs and an economic burden in the healthcare system. Our data can help to define the requirements for a digital data management system for medical data in Germany.

## 2. Material and Methods

### 2.1. Ethics Approval and Patient Recruitment

The study was conducted according to the guidelines of the Declaration of Helsinki and was approved by the Ethics Committee of Hannover Medical School (No. 8788_BO_K_2019—Amendment 10 May 2022).

### 2.2. ED Assessment

We included all neurological patients (n = 272) who were admitted to the ED during a total of 60 days of assessments. All patients were included regardless of the time of their admission (day or night, 24/7). The mode of admission was documented (via ambulance, with medical referral, self-admission). One investigator conducted the assessments during the day, and for patients who were admitted in the evening or at night, their responsible doctor gave the relevant information to our investigator the next morning. Nevertheless, a few patients could not be recorded as information on their ED visit could not be sufficiently reconstructed.

First, we conducted a short survey to assess the availability of important medical data upon arrival in the ED. To determine the potentially most relevant data, we defined three categories of information in advance: 1. *Pre-existing diseases*, 2. *Pre-existing medication*, and 3. *Allergies*. When assessing the data availability of these categories, we differentiated between 1. *Information complete*, 2. *Information incomplete*, and 3. *Information not available at all*.

We defined a “critical data score” as incomplete information in two or more categories and/or missing information in one or more categories. In these cases, we expected to see complications due to reduced data availability in the ED. Based on the initial ED survey, the patients were divided into the groups “good data score” (information in all three categories complete) and “critical data score”, and the patients remained in these groups for the rest of the data analysis. We decided to stratify patients with incomplete data in one category (n = 18) into neither the “good data score” or “critical data score” group. Incomplete information, e.g., in the category of preexisting medication can complicate the ED care and can thus not be considered a “good data score”. However, we wanted to include only patients in the “critical data score” group amongst whom data-associated complications would be expected, and we did not necessarily expect them when information in only one category was incomplete. These 18 patients were included in the analysis of all patients.

In patients with initially incomplete data, information about diagnoses and medication was added to the patients’ dataset if available at a later time to have complete demographic and clinical information (e.g., multimorbidity, polypharmacy). Then, we investigated how smoothly the emergency care of the patients was performed, using a questionnaire completed by the responsible doctors. We asked whether additional phone calls were necessary and if delays or complications during acute care were observed due to missing data. Another aspect we investigated was whether a healthcare proxy and/or advance directive were of relevance in the ED setting.

### 2.3. Inpatient Assessment

All participants in inpatient care gave written informed consent prior to inclusion. Only patients who were admitted via the ED and were part of the ED assessment were included in the inpatient analysis (n = 142).

For the inpatient assessment, the responsible doctors were asked about delays and complications during the hospital stay (via a questionnaire) by one investigator. We assessed whether the advance directive and/or healthcare proxy were relevant during the hospital stay. Finally, we wanted to know whether the hospitalization was prolonged or could have even been avoided if all medical information had been available upon ED admission.

### 2.4. Data Analysis

Data are displayed as mean ± standard deviation, as total numbers/fractions or as percentages. Fractions of patients with complications or other events were compared using a Fisher’s exact test. The age of patients in the “critical data score” and “good data score” groups was compared using a non-parametric *t*-test (Mann–Whitney U test). *p*-values are shown only for significant comparisons. Statistical analysis was performed using the GraphPad Prism Software (version 10.2.0, Boston, MA, USA). Due to the exploratory nature of this study, no correction for multiple comparisons was applied.

## 3. Results

### 3.1. Data Availability upon ED Admission (N = 272)

**Figure 1:**
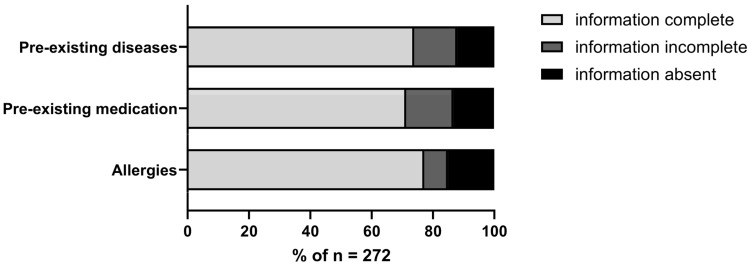
**Data availability in the neurological emergency department: good data score** (data in all 3 categories complete) n = 180 (66%), **critical data score** (data in ≥2 categories incomplete and/or absent in ≥1 category) n = 74 (27%), **all data absent** (included in the critical data score group) n = 30 (11%), data incomplete in one category (n = 18).

A total of 66% of the patients admitted to our ED presented with complete medical information (diagnoses, medication, allergies) and were part of the “good data score” group (Figure 1). In 11% of the patients, no information about diagnoses, medication and allergies was available at all (Figure 1). Altogether, 27% of the patients were stratified into the “critical data score” group (Figure 1).

### 3.2. Patient Characteristics (N = 272)

The patients admitted to the ED had a mean age of 59.6 ± 21.3 years, and 50% were male. Amongst all the patients, 58% were multimorbid (≥three chronic diseases), and 38% were under polypharmacy (defined as ≥ five chronic medications) (Table 1). Amongst all the patients, 53% were admitted during standard working hours (between 8 am and 4 pm), and 53% came via ambulance (Table 1). Patients in the “good data score” group were significantly younger than patients stratified into the “critical data score” group (57.3 ± 215 vs. 65.6 ± 18.4, *p* = 0.0046). Multimorbidity was more prevalent amongst the patients with incomplete data (52/71 vs. 89/177, *p* = 0.0011). Significantly more patients with a “critical data score” were admitted to the ED via ambulance (57/3 vs. 77/180, *p* < 0.0001), while more patients with complete data admitted themselves (70/180 vs. 10/73, *p* < 0.0001).

### 3.3. Complications in Emergency Care (N = 272)

Doctors in the ED had to make phone calls in 32% of all cases, and in 57% of cases with a critical data situation, which was significantly more than in the “good data score” group (42/74 vs. 40/180, *p* < 0.0001) (Table 2). A relevant delay in the acute care was documented for 20% of the patients with a critical data situation (15/74 vs. 4/180, *p* < 0.0001) (Table 2). In general, delays between 5 and 240 min were observed with a median time of 45 min per delay. As consequence, a delay in the application of thrombolysis therapy for ischemic stroke was reported, which may result in a worse clinical outcome for the patient. In addition, a delay in hemodialysis was observed for a patient with missing medical information.

Additionally, unnecessary diagnostic procedures were applied in 5% of the patients with a “critical data score” (4/74 vs. 0/180, *p* = 0.0068) (Table 2). Amongst these, two cases of lumbar puncture, cerebral imaging (computer tomography and magnetic resonance imaging), including application of contrast medium, laboratory and urological diagnostics, were documented.

### 3.4. Complications in Inpatient Care (N = 142)

Doctors in inpatient care reported a persisting influence of the initial data situation in the ED on the care of the admitted patients. Phone calls from the ward were necessary for 59% of the patients with a critical data situation in the ED and for only 25% of patients with a “good data score” (23/39 vs. 23/92, *p* = 0.0003) (Table 3). A time effort for additional data acquisition between 10 and 180 min was reported. Even in inpatient care, unnecessary diagnostic procedures were performed (8%) because medical data were still missing (Table 3). Again, cerebral imaging, laboratory diagnostics, urological diagnostics, and also a gastroscopy and a coloscopy, were documented as examples. Doctors in inpatient care stated that the inpatient stay of patients with a critical data situation in the ED was prolonged in 8% of the cases due to the missing data (Table 3). Amongst the patients who came to the ED with complete data in the categories investigated, doctors reported a prolonged inpatient stay due to missing data in only 1% of the cases (Table 3). Finally, for 5% of patients with an initially critical data situation, doctors stated that the entire inpatient stay could have been avoided if all information had been available upon ED admission (0% in cases with complete medical information upon ED admission) (Table 3).

### 3.5. Patient Autonomy (N = 257)

Especially in older and multimorbid patients, questions about the patient will can arise in acute and inpatient care when dealing with severe diseases. We investigated how often the patient will or legal representatives played a role in the care of our neurological patients. Amongst all the patients in our neurological ED, 24% stated that they had an advance directive and 25% that they had a healthcare proxy/legal representative (Table 4). Of these patients, only 16% brought these documents to the ED admission (Table 4). Interestingly, slightly more patients in the “critical data score” group reported having a healthcare proxy, which could be due to the higher age in that group (25/70 vs. 36/170, *p* = 0.0227). Doctors in the ED reported that the advance directive or healthcare proxy would have been needed in the acute care of the patients in 15% of the cases with a “critical data score” and in only 6% of patients with a “good data score” (11/74 vs. 10/180, *p* = 0.0222) (Table 4). Mainly, this was due to decisions regarding invasive procedures, e.g., intensive-care treatment with artificial ventilation for severely ill patients. In these cases, information about the patient will or the input of legal representatives are crucial to achieve a decision that is compliant with the patient’s wishes. In inpatient care, this topic was even more relevant as doctors reported needing the advance directive or healthcare proxy in 49% of cases with a “critical data score” (19/39 vs. 19/92, *p* = 0.0028) (Table 4).

## 4. Discussion

The number of geriatric, multimorbid patients treated with multiple drugs (polypharmacy) is increasing in various societies due to demographic developments [2,3,4]. Worldwide, more than half of the population at an age of 60 or older is multimorbid, and in Europe, multimorbidity has reached a prevalence of 39.2% in the overall population [2]. At the same time, multimorbid patients are complex to handle in emergency situations because chronic conditions and medications influence what diagnostics and treatments are suitable for an individual, which underlines that the medical history is crucial for emergency care [1]. We postulated that the absence of this information in the ED might compromise patient safety. Our results show that multimorbidity was very frequent amongst neurological ED admissions (58%). Missing or incomplete medical data were reported for 34% of multimorbid patients (n = 154) and for 27% of all patients (n = 272) upon arrival at our ED. This caused a potentially dangerous delay in the acute care of 10% of multimorbid and 8% of all patients. We even recorded one case of treatment delay for a very time-sensitive stroke treatment (i.v. thrombolysis), which can result in a worse clinical outcome for the patient. The duration of boarding in the emergency department has been linked to negative patient outcomes, including reduced satisfaction and increased inpatient mortality rates (mortality of 2.5% in patients boarding less than two hours vs. 4.5% in patients boarding 12 h or more) [10]. A study analyzing malpractice claims for missed and delayed diagnoses found that failure to obtain an adequate history (42%) as well as patient-related factors (46%) (non-adherence, atypical clinical presentation or complicated medical history) were very common amongst the reasons for insufficient care resulting in patient harm [11].

Patient safety-compromising events like unnecessary invasive diagnostics or wrong judgements were reported in 5% of the cases in our sample. A lumbar puncture, for instance, has risks that are only appropriate to take if there is a clear indication for the examination. We recorded two cases of unnecessary lumbar punctures in our sample. A previous study showed that imaging overuse is a common problem and especially relevant in older and multimorbid patients [12]. One reason for reimaging a patient was transfer to another hospital with inadequate data transmission in a time-sensitive situation [12]. Our results are in line with those previous reports and support the notion that (digital) availability of medical data in hospitals is necessary to avoid non-indicated diagnostic procedures which can potentially endanger the affected patients.

As long as no comprehensive management system for medical data is available, stratifying patients by their data availability according to our “critical data score” and “good data score” group might be a quick and useful tool to identify patients at risk for missing data-associated complications. This can alert the treating physicians to be careful with decisions regarding these patients’ diagnostic and therapeutic procedures in the ED. The score is easy to assess and apply, as information on pre-existing diseases, medication and allergies has to be obtained in the ED anyhow.

However, it has to be noted that we also observed some delays but very few unnecessary diagnostic procedures in the group with a “good data score” (Table 2 and Table 3). We can hypothesize that missing information about legal representatives and the patient will also force doctors to make telephone calls resulting in delays in acute and inpatient care.

An interesting observation in our study was that patients with a “critical data score” were admitted via ambulance significantly more often than patients with complete medical data. This can possibly be due to the fact that the patients with incomplete data were older and more often multimorbid, which might result in more acute hospital admissions. But this also hints at the fact that the rescue services can apparently not help to gather important medical information in emergency cases.

Another important aspect that our study put a focus on is patient autonomy. Especially for chronically and severely ill patients, the expression and execution of their will is very important. In emergencies, it is obviously difficult to assess the patient will, especially if the patients cannot report it themselves and the corresponding documents are not available. Our results demonstrate that about 25% of the patients reported having an advanced directive, but only 16% of these patients (4% in total) brought their patient will to the ED upon admission, although it was needed in the acute situation in 8% of all cases. Regarding only the patients at an age of ≥ 80 years, 57% had an advanced directive, but only 19% of these brought it to the ED admission. This in line with previous studies outlining the availability of patient wishes as a problem in respecting patient autonomy in emergencies [13,14]. About half of the older adults admitted to an American ED reported having advance directives or healthcare proxies, while only about 4% of these patients had them properly documented in their electronic medical record [15]. Thus, the advance planning of older patients is prevalent in a large proportion of patients, but the availability of the data prevents its use in the emergency setting. This could be optimized if these aspects were considered in the design of a digital data management system. Patients’ wishes appear to be even more relevant in the inpatient setting (required in 29% of inpatients in our study). The responsible doctors in our investigation most likely had to spend additional time gathering information on the patient will, which could have been avoided if these data had been digitally available.

Healthcare costs for multimorbid individuals are high and increasing with the number of chronic conditions; thus, they pose a significant (economic) challenge to healthcare systems worldwide [9,16,17]. Specifically, annual healthcare costs for an individual with one chronic condition were USD 7172 in an American study, whereas costs for individuals with three or more chronic conditions increased to USD 32,498 on average [18]. The annual attributable costs of multimorbidity in older adults in Canada were reported to range between CAD 1026 and CAD 3831 [17]. In 2009/2010, 24.4% of the population of Ontario had at least two chronic medical conditions, but these individuals accounted for about 67.9% of the total allocable healthcare costs [17].

A retrospective study of 13,460 patients in a tertiary care teaching hospital reported that the length of inpatient stays and the costs increased if there was a relevant delay in the ED process [19]. A relevant delay was observed in 11.6% of all admitted patients in this study, and this led to 12.4% longer inpatient stays (2183 additional inpatient days) and 11.0% greater costs for the hospital stay [19]. Similar results were obtained by Singer et al. who observed a significantly increased length in the inpatient stay in patients who remained in the ED for more than 24 h after the decision to admit them, compared to patients who were admitted to a ward within two hours [10]. It has also been shown that, in America, electronic medical records can reduce the length of stay in the ED as well as the diagnosis/treatment time [20]. In the study, EDs with a fully functional electronic medical record system achieved a 22.4% shorter length of stay in the ED and 13.1% lower diagnosis/treatment time than EDs with minimal or no electronic medical records [20]. This is of great importance as a prolonged stay, especially overnight, increases the mortality in older patients [21]. The data presented here show that missing medical data are an important reason for delays in EDs in Germany.

In general, multimorbidity is also associated with more frequent hospital admissions [7,8,16]. Furthermore, avoidable hospitalizations are more common in patients with multiple chronic conditions and also result in a financial burden for the healthcare system [9]. Medicare beneficiaries with at least four chronic conditions were 99 times more likely to be admitted to the hospital for problems that could have been resolved in ambulatory care than beneficiaries without chronic conditions [9]. We also recorded avoidable hospitalizations due to a lack of medical information in our cohort. A data management system might prevent these hospitalizations, thereby reducing healthcare costs.

We observed that the missing medical data required the ED doctors to spend time on acquiring this information (e.g., via phone calls). Since this is “expensive” time, every phone call related to missing medical data adds to the economic healthcare burden.

Digital data management systems for medical data can be a solution to some problems addressed in this study. In Europe, implementation of electronic medical records is handled diversely by different countries [22]. Denmark is mentioned as an example for the broad use of an electronic data management system in the healthcare sector [22,23]. In Germany, no comprehensive data management system for medical data has been established so far [24]. An electronic medical record system is currently under development and implementation in Germany, but there is still room for input on how a digital healthcare system should be equipped [24]. While electronic medical records are expected to improve emergency care, there are also reports implying that the use of electronic medical records increases the time required for documentation [22]. However, several studies have shown that admission and readmission rates are reduced when electronic medical records are used [22]. The effect of digital data management systems on acute and inpatient care should be the objective of future studies.

## 5. Conclusions

This study supports the implementation of a comprehensive digital data management system for medical information optimizing acute care for all, especially older and multimorbid patients. The results show that basic medical data like diagnoses, medication and allergies are needed to make good decisions, particularly in emergency care, and that their absence often results in delays because doctors have to acquire these data, even in time-sensitive situations. A digital medical record would be helpful if this information were complete and up to date, as false or missing information represents a potential hazard. Our study also reveals that it can be useful to implement advance directives and healthcare proxies into such a digital tool, as these documents were proven to be of a high relevance in neurological care. This aspect should be considered when designing medical data management systems.

## Figures and Tables

**Table 1:** Patient characteristics; n may vary due to data absence.

Neurological Patients in the Emergency Department	All Patients n = 272	Good Data Score n = 180	Critical Data Score n = 74
Age	59.6 ± 21.3	57.3 ± 21.5	65.6 ± 18.4 ** *p* = 0.0046
Sex female	50.4%	99/180 (55.0%)	32/74 (43.2%)
Multimorbidity (≥5 chronic diseases)	57.9%	89/177 (50.3%)	52/71 (73.2%) ** *p* = 0.0011
Polypharmacy (≥ 3 chronic medications)	38.2%	57/174 (32.8%)	31/67 (46.3%)
Language barrier	6.7%	8/171 (4.7%)	8/73 (11.0%)
Admission during regular working hours	53.3%	97/180 (53.9%)	35/74 (47.3%)
Admission outside of regular working hours	46.7%	83/180 (46.1%)	39/74 (52.7%)
Admission via ambulance	52.9%	77/180 (42.8%)	57/73 (78.1%) **** *p* < 0.0001
Admission with medical referral	15.4%	33/180 (18.3%)	6/73 (8.2%)
Self admission	31.6%	70/180 (38.9%)	10/73 (13.7%) **** *p* < 0.0001

** *p* < 0.005, **** *p* < 0.0001. Age was compared using a Mann–Whitney U Test; fractions were compared using a Fisher’s test.

**Table 2:** Complications in Acute Care.

	All Patients n = 272	Good Data Score n = 180	Critical Data Score n = 74
Telephone calls	32.0%	40/180 (22.2%)	42/74 (56.8%) **** *p* < 0.0001
Unnecessary diagnostics	1.5%	0/180 (0%)	4/74 (5.4%) ** *p* = 0.0068
Relevant delay in diagnostics/therapy	7.7%	4/180 (2.2%)	15/74 (20.3%) **** *p* < 0.0001
Wrong judgement	1.5%	0/180 (0%)	4/74 (5.4%) ** *p* = 0.0068

** *p* < 0.005, **** *p* < 0.0001; fractions were compared using a Fisher’s test.

**Table 3:** Complications in Inpatient Care.

	All Patients n = 142	Good Data Score n = 92	Critical Data Score n = 39
Telephone calls	35.2%	23/92 (25.0%)	23/39 (59.0%) *** *p* = 0.0003
Unnecessary diagnostics	3.5%	1/92 (1.1%)	3/39 (7.7%)
Inpatient stay extended	4.2%	1/92 (1.1%)	3/39 (7.7%)
Inpatient stay could have been avoided	2.1%	0/92 (0%)	2/39 (5.1%)

Extension and unnecessary inpatient stays were due to initially missing data. *** *p* < 0.0005; fractions were compared using a Fisher’s test.

**Table 4:** Patient Autonomy.

	All Patients n = 257	Good Data Score n = 170	Critical Data Score n = 70
Pre-existing advance directive	24.1%	36/170 (21.2%)	23/70 (32.9%)
Pre-existing healthcare proxy	24.9%	36/170 (21.2%)	25/70 (35.7%) * *p* = 0.0227
Pre-existing document brought to the ED	15.6%	6/36 (16.7%)	4/25 (16.0%)
AD/HCP relevant in acute care	8.1%	10/180 (5.6%)	11/74 (14.9%) * *p* = 0.0222
AD/HCP relevant in inpatient care	28.9%	19/92 (20.7%)	19/39 (48.7%) * *p* = 0.0028

Abbreviations: Advance directive (AD), healthcare proxy (HCP), emergency department (ED). * *p* < 0.05; fractions were compared using a Fisher’s test.

## Data Availability

The raw data supporting the conclusions of this article will be made available by the authors on request.
